# [Retracted] FAM172A induces S phase arrest of HepG2 cells via Notch 3

**DOI:** 10.3892/or.2023.8554

**Published:** 2023-04-24

**Authors:** Zhiqiang Feng, Hongqi Li, Shunai Liu, Jun Cheng, Guoan Xiang, Jinqian Zhang

Oncol Rep 29: 1154–1160, 2013; DOI: 10.3892/or.2013.2235

Following the publication of the above article, a concerned reader drew to the Editor's attention that the western blots featured in Figs. 1G, 2B, 3B and 4E contained groupings of bands that were markedly similar in appearance, both within the same gel slices and comparing across different gel slices between the figures in the case of Figs. 3 and 4.

After having conducted an internal investigation of this matter, the Editor of *Oncology Reports* has judged that the anomalous groupings of data were too extensive that their apperance could have been attributed to pure coincidence. Therefore, the Editor has decided that this article should be retracted from the publication on the grounds of an overall lack of confidence in the data. After having been in contact with the authors of this study, they accepted the Editor's decision to retract this article. The Editor sincerely apologizes to the readership for any incovenience caused, and we thank the reader for bringing this matter to our attention.

